# Strain Balanced AlGaN/GaN/AlGaN nanomembrane HEMTs

**DOI:** 10.1038/s41598-017-06957-8

**Published:** 2017-07-25

**Authors:** Tzu-Hsuan Chang, Kanglin Xiong, Sung Hyun Park, Ge Yuan, Zhenqiang Ma, Jung Han

**Affiliations:** 1 Department of Electrical and Computer Engineering, University of Wisconsin-Madison, Madison, 53706 United States; 2 Department of Electrical Engineering, Yale University, New Haven, 06511 United States

## Abstract

Single crystal semiconductor nanomembranes (NM) are important in various applications such as heterogeneous integration and flexible devices. This paper reports the fabrication of AlGaN/GaN NMs and NM high electron mobility transistors (HEMT). Electrochemical etching is used to slice off single-crystalline AlGaN/GaN layers while preserving their microstructural quality. A double heterostructure design with a symmetric strain profile is employed to ensure minimal residual strain in freestanding NMs after release. The mobility of the two-dimensional electron gas (2DEG), formed by the AlGaN/GaN heterostructure, is noticeably superior to previously reported values of many other NMs. AlGaN/GaN nanomembrane HEMTs are fabricated on SiO_2_ and flexible polymeric substrates. Excellent electrical characteristics, including a high ON/OFF ratio and transconductance, suggest that III-Nitrides nanomembranes are capable of supporting high performance applications.

## Introduction

Single crystal semiconductor nanomembranes (NM) have gained much attention in recent years with promising applications, including heterogeneous integration and flexible electronics^1–4^. For heterointegration of semiconductors, NMs can be transferred onto and bonded with different host substrates^1–3^. For flexible electronic devices, semiconductors need to be fashioned into two-dimensional (2D) forms with thickness much below micrometer (µm) range^4^. Indeed several electronic materials have been prepared into NMs including III-V semiconductors^5, 6^, Si^7–9^, and amorphous oxides^10^, polymers^11^, 2D materials like graphene^12, 13^ and transition metal dichalcogenides^14–16^. Among these materials, inorganic Si and III-V nanomembranes are appealing because of compatibility with conventional processing without the compromise in device performance. Semiconductor III-V NMs are prepared by selective wet etching of sacrificial layers^17^, while Si NM can be fabricated in similar way by etching of the buried oxide layer of SOI wafers^18^.

III-Nitride is wide band gap semiconductor known for its optoelectronic properties. AlGaN/GaN HEMTs have demonstrated their advantages in high power, radio frequency and energy-efficient transistors, working in harsh environments^19–21^. However, their application in the form of NM has not been explored. One particular reason is that III-Nitride is chemically inert with no wet chemical etching at room temperature^22^; selective etching of GaN has been a challenging task.

Significant efforts have been devoted to obtain III-Nitride epilayers in a freestanding form^23–25^. Excimer-laser based lift off has been developed to separate GaN from sapphire by thermally decomposing the GaN region near substrate interface^23^. For samples grown on silicon substrates, both wet^24^ and gaseous etching^25^ have been developed to selectively remove the silicon substrates; several micrometers-thick AlGaN high electron mobility transistors (HEMTs) have been transferred onto plastic substrates^24^. All of these approaches produce films with a thickness of (much) greater than 1 μm. For applications requiring conformity and flexibility, nanomembranes are necessary because of their much reduced flexural rigidity^26^.

Recently a conductivity-selective electrochemical etching process has been reported^27^. This process has been employed to fabricate photonic structures and nanomembranes^28, 29^. GaN light emitting diodes (LEDs)^30^ and enhanced-mode metal-oxide-semiconductor (MOS) transistors on ridge substrate have been demonstrated^31^. Compared to the MOS transistors, AlGaN/GaN HEMTs employing high-mobility two-dimensional electron gas (2DEG) are more desirable for performance-sensitive electronics. However, fabrication of AlGaN/GaN heterostructure NM is more challenging. The feasibility of NM HEMTs has not been investigated yet.

In this paper, we report the preparation of a 300 nm-thick AlGaN/GaN/AlGaN heterostructure NM and NM-based HEMTs on different substrates with high electron mobility. Large area, freestanding, and strain-balanced AlGaN/GaN/AlGaN NM has been prepared from III-Nitrides epitaxial layers on sapphire substrate. The crystalline quality of the NM is comparable to epitaxial GaN with dislocation density of 5 × 10^8^ cm^−2^. The mobility of the 2DEG at the top AlGaN/GaN interface is about 800 cm^2^/V s. HEMT devices have been prepared on SiO_2_/silicon substrates and flexible PET films, with low leakage current and a ON/OFF ratio of 10^7^.

## Results

**Figure 1:**
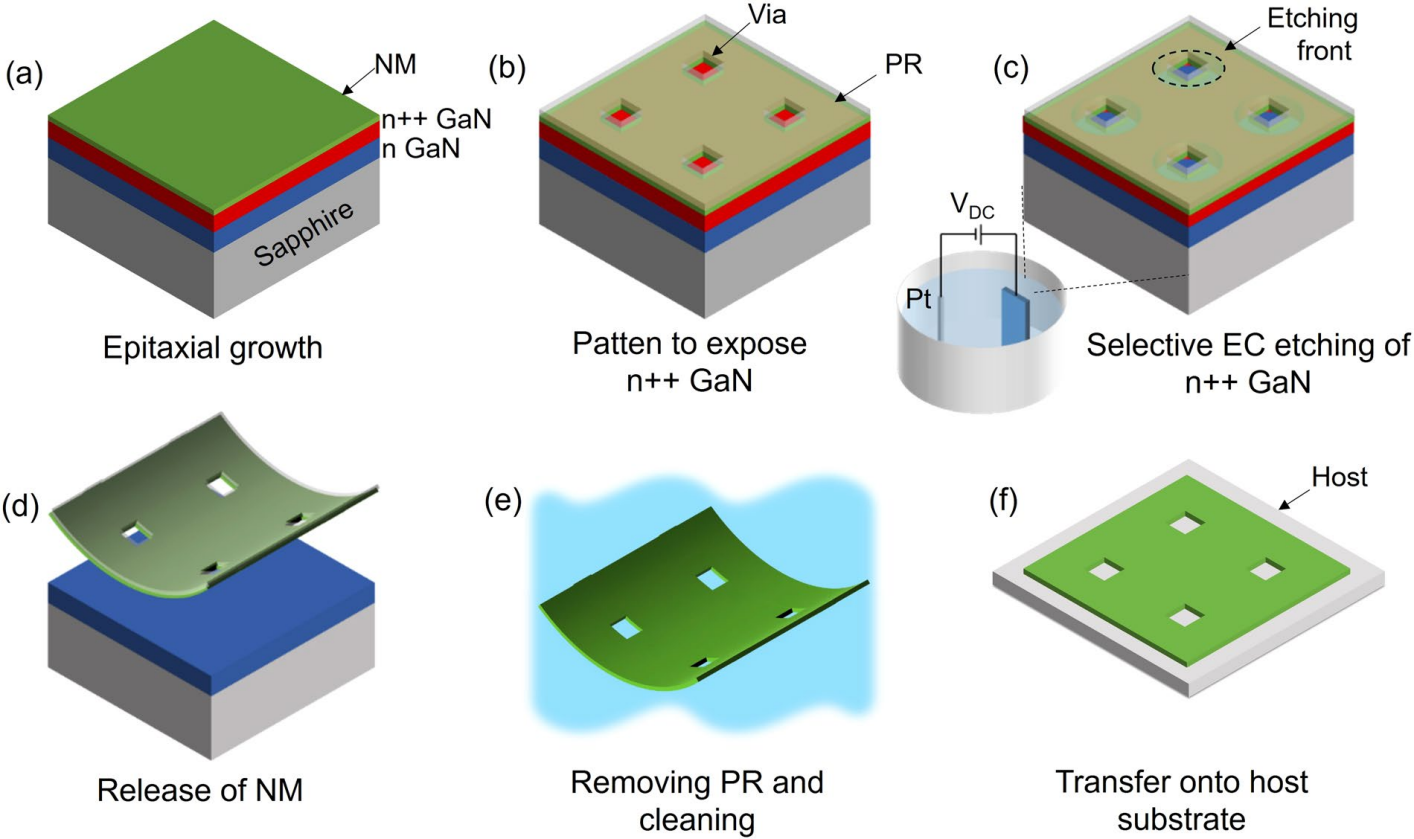
Fabrication procedure of III-Nitride NM. (**a**) Epitaxial growth of NM to be lift-off with an underneath n++ GaN layer, (**b**) photolithograph and dry etching to expose n++ GaN sidewalls with arrays of vias, (**c**) selective undercut etching of n++ GaN by electrochemical etching, (**d**) separation of NM from epitaxial wafer after full undercut, (**e**) removal of photoresist and cleaning the NM, (**f**) transfer the freestanding NM onto a host substrate.

The process flow to fabricate the III-Nitride NM is illustrated in Fig. 1(a)–(f) sequentially. The preparation of NM starts with the epitaxial growth of III-Nitride on sapphire substrate by metal-organic chemical vapor deposition. The NM structure to lift-off is grown on top of a 2 µm GaN buffer layer with a heavily doped n++ GaN layer serving as a sacrificial layer. The lift-off of NM is based on the conductivity-selective electrochemical (EC) etching of the n++ GaN sacrificial layer laterally. (Figure S1) The EC etching behavior of GaN depends on its doping level as well as the bias voltage^32^. Voltage is typically held constant throughout the etching. The etching rate ranges from 1 to 50 μm/min, varying with electrolytes and bias voltage. To achieve large-area (~cm^2^) NM within a reasonable time, the epitaxial wafer is lithographically patterned with a 2-D array of via holes spaced at 50 to 100 μms to expose n++ GaN as shown in Fig. 1(b), using Cl-based dry etching. In Fig. 1(c), the n++ GaN is etched from the edge of via windows in an isotropic way while NM and other layers stay intact. Etching proceeds with the advancing and eventually coalescing of the etching fronts; then NMs will separate from the substrate and become suspended in electrolyte solutions as shown in Fig. 1(d). We note that dry-etching masks, either photoresist or SiO_2_, can not only prevent vertical etching through the NM surface but also provide mechanical support during and after electrochemical etching. Once the EC etching is finished with the release of NMs, the floating NMs are gently rinsed in DI water and solvent to remove chemical residuals, and transferred onto host substrates.

**Figure 2:**
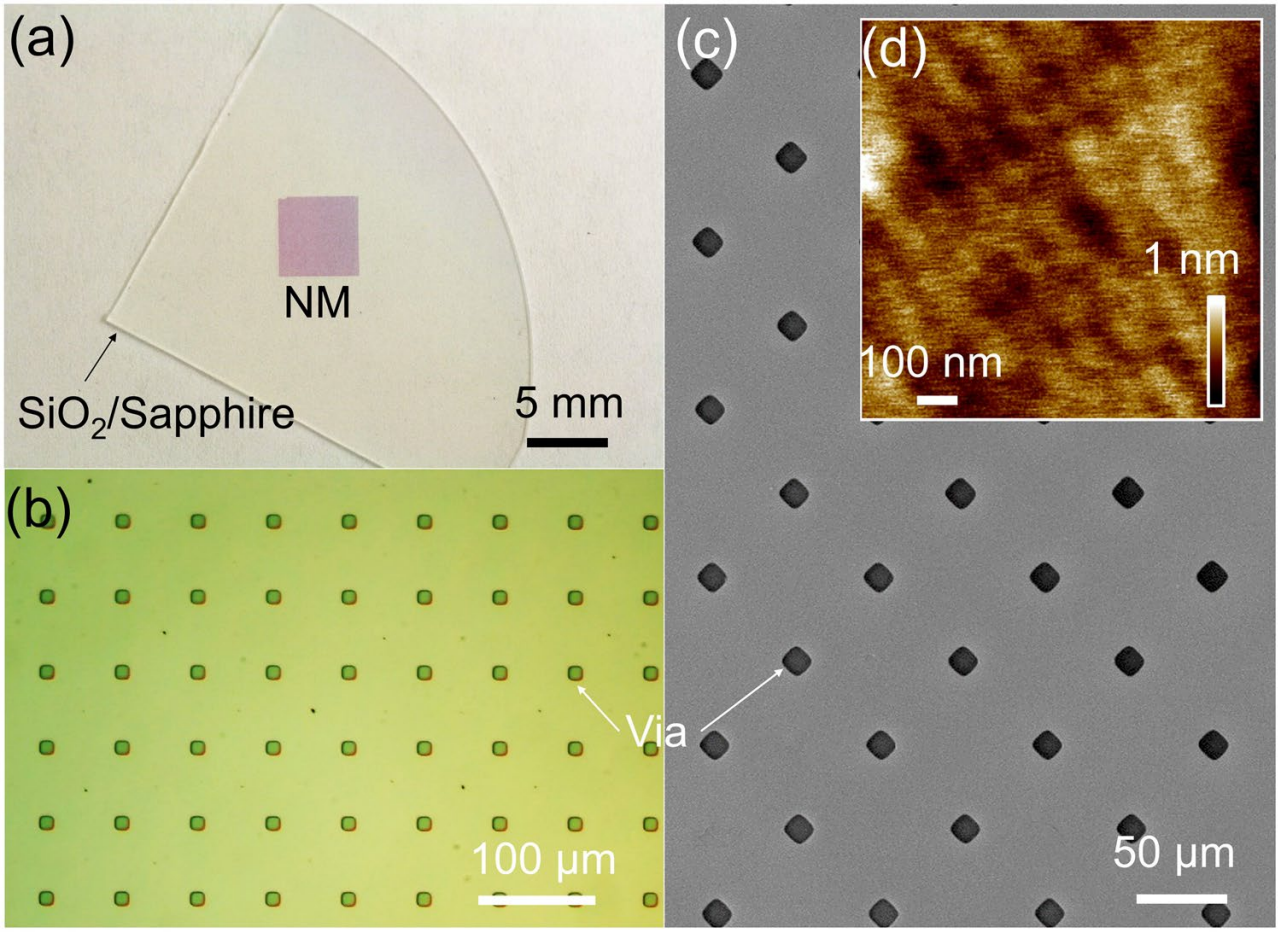
(**a**) An AlGaN/GaN/AlGaN NM on SiO_2_/Sapphire, (**b**) Microscopy image of a NM on SiO_2_/Si with diagonal spacing of 100 µm, (**c**) and (**d**) SEM and AFM images of the Ga-polar surface of a NM, respectively.

There are two challenges in the fabrication of AlGaN/GaN NMs. First, the desirable goal of creating 2DEG at the AlGaN/GaN interface introduces additional electrical current-flowing pathways in the sample during EC etching. Given that EC etching, employed to laterally undercut n++ GaN and release the nanomembranes, is conductivity selective, the presence of the highly conductive 2DEG leads to unintentional and parasitic etching at the AlGaN/GaN interface in addition to etching of the sacrificial layer. (Figure S2(a)) A Fe-doped highly resistive GaN interlayer is therefore employed between the sacrificial layer and the 2DEG as a current-blocking layer^33^. Considering the memory effect of Fe during growth and its influences on 2DEG, the thickness and doping level of the Fe-GaN layer need to be optimized. A Fe-GaN (25 nm, ~1 × 10^18^ cm^−3^) layer is found to prevent vertical current flow into the top AlGaN/GaN interface and curb the undesirable parasitic etching effectively without degrading the 2DEG much.

The second issue in fabricating AlGaN/GaN NMs is the presence of residual heteroepitaxial strain in the NMs after release. Unlike the well-known AlAs/GaAs system where the entire ternary alloy is essentially lattice-matched, the AlN-GaN binary end compounds have a lattice mismatch of 2.4%. Management of residual strain in the composite NM system becomes very critical. By balancing the force and bending moment in a two-layer system, one arrives at Stoney formula which interrelates parameters including stress, thicknesses, and the resultant radius of roll-ups^34^. Applying Stoney’s equation to the specific AlGaN/GaN nanomembrane system, one can derive an empirical expression, assuming the thicknesses of AlGaN and GaN to be, respectively, 25 and 150 nm, that the radius of curvature as a function of Al% to be 8.9 µm/x_Al_. (Equation S3) The use of an AlGaN barrier with an Al composition of 25% (x_Al_ = 0.25) would result in a rolling up of nanomembrane with a radius of curvature of only 36 μm! Indeed, such a rollup behavior has been observed in initial attempt. (Figure S2(b)) A double heterostructure (DH) design of Al_0.25_Ga_0.75_N/GaN/Al_0.25_Ga_0.75_N with a symmetric strain profile is adopted such that a free-standing membrane will remain strain balanced and flat^9^.﻿

Images of strain-balanced NMs from Al_0.25_Ga_0.75_N (25 nm)/GaN(250 nm)/Al_0.25_Ga_0.75_N (25 nm) DH design and with Fe-GaN layer are shown in Figure 2(a)–(c). The thickness of the sandwiched GaN layer is adjusted to keep the strain in AlGaN layers thus the 2DEG density. With a total thickness of 300 nm, the NMs are conformal to and adhere to almost any surfaces by van der Waal’s force. The area of NM in Figure 2(a) is 0.5 × 0.5 cm^2^. Larger NM of 1 × 1 cm^2^ can also be fabricated. The front surface is protected during the EC etching, thus a smooth surface is expected, and confirmed by AFM measurement in Figure 2(d).

**Figure 3:**
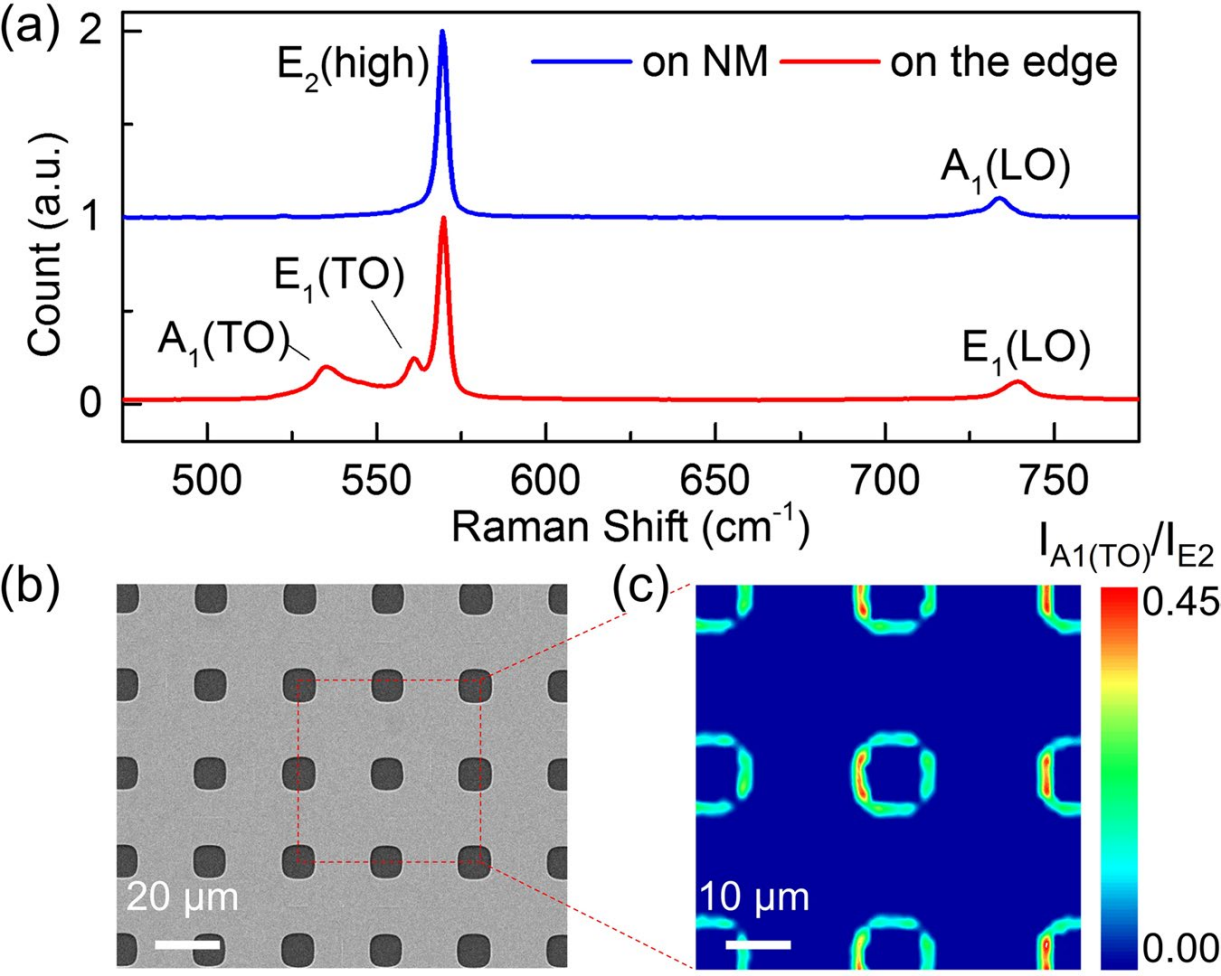
(**a**) The Raman spectrum of DH NM with the exciting laser spot on the NM and on the edge of NM. (**b**) SEM image of an N-polar NM with diagonal via spacing of 25 µm (**c**) Spatial mapping of intensity ratio of the A_1_ (TO) to E_2_ (high) peak. The scanning area is 60 × 60 µm^2^, indicated by red square in (**b**).

Micro-Raman is used to measure the state of strain in a freestanding DH NM using 532 nm laser as excitation source. The spectra are shown in Fig. 3(a), with exciting laser on the NM and on the edge of NM, respectively. In both cases, majority of Raman scattering signal comes from the GaN layer due to its larger thickness. It is noticed that on the NM, there are only E_2_ (High) and A_1_ (LO) peaks of GaN visible, which is signature of single crystal wurtzite GaN^35, 36^. In contrast, A_1_ (TO) and E_1_ (TO) peaks are prominent on the edge of the NM, due to coupling of laser into the plano-waveguide like AlGaN/GaN/AlGaN NM, and subsequent scattering. The Raman spectra within NM is distinctive from single crystal nanoporous GaN film where all the above peaks appear together^37^. Figure 3(b) and (c) show SEM image of an N-polar NM and the spatial intensity-ratio mapping the A_1_ (TO) to E_2_ (high) peak on the NM. The A_1_ (TO) is only observable surrounding the edges of the vias. The absence of A_1_ (TO) peak within NM region indicates that there is no obvious damage such as vertical etching or cracks within the NM that is well preserved during the electrochemical etching and transfer process.

With 0.6% lattice mismatch between Al_0.25_Ga_0.75_N and GaN, there is noticeable strain distribution in the sandwich structure. Namely, GaN is compressively strained while AlGaN is stretched in lateral direction. Assuming no plastic relaxation of mismatched strain and adopting the same Young’s modulus for AlGaN and GaN, the in-plane strain of GaN can be estimated by the strain partition rule to be 0.1%^34^. The strain of the GaN layer can also be measured from the shift of E_2_ peak of 569.4 cm^−1^ compare to freestanding GaN of 567.4 cm^−1^ ^30^. The value is 0.11% according to relative Raman shift of 2.0 cm^−1^ (Δω_E2_ = Δσ_a_ × *k*_a_, *ϵ*_a_ = Δσ_a_/M, where Δω_E2_, Δσ_a_ is Raman shift, in-plane stress, respectively; the value for coefficient *k*_a_, biaxial modulus M is 4.2 cm^−1^/GPa, 449.6 GPa)^38, 39^. The agreement between calculated and measured results suggests the AlGaN and GaN in the NM share the coherent lattice.

**Figure 4:**
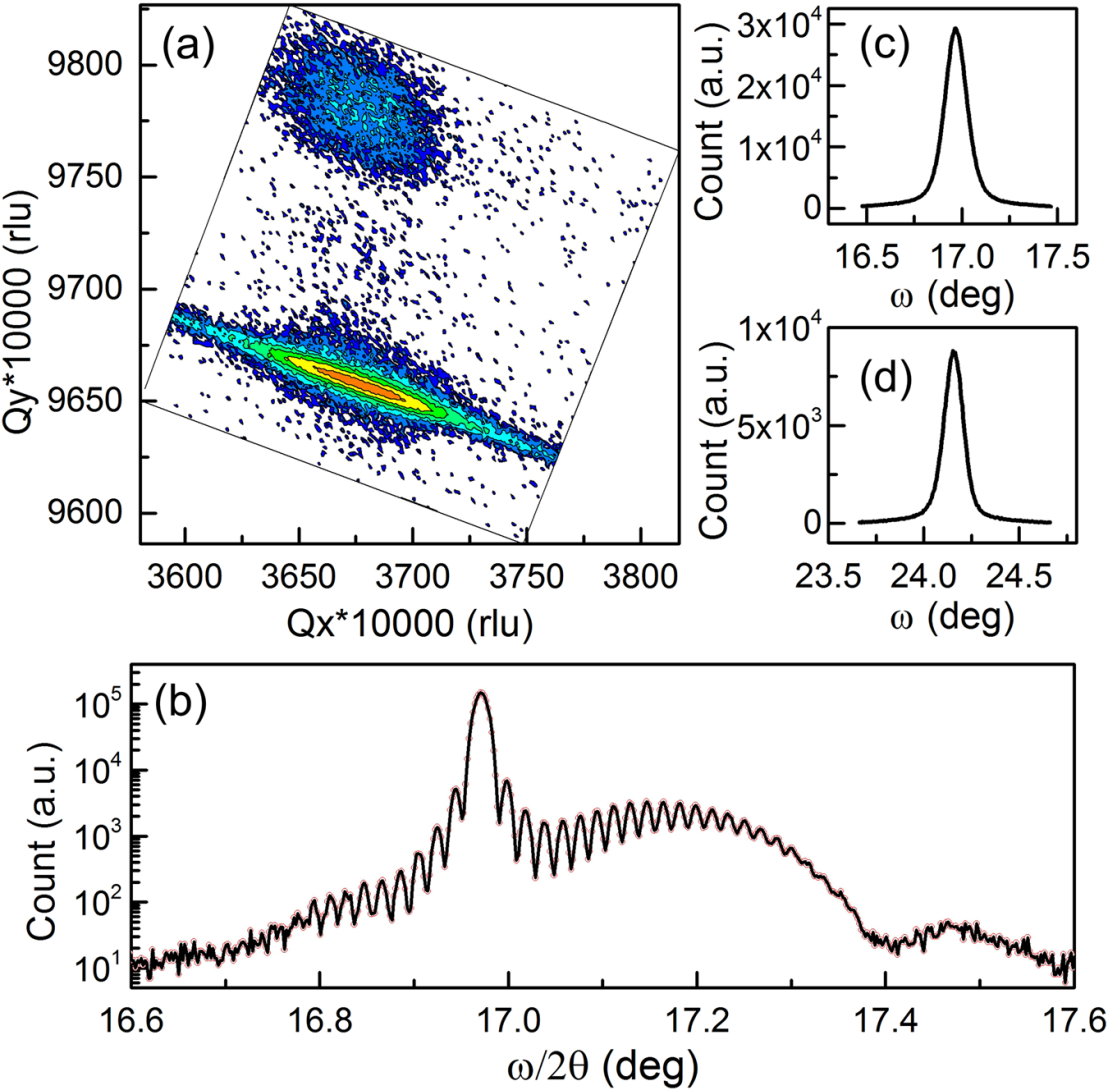
High resolution X-ray diffraction of DH NM on SiO_2_/Si. (**a**) The (105) reciprocal space mapping. (**b**) (002) ω/2θ scan. (**c**) (002) rocking curve of GaN. (**d**) (102) rocking curve of GaN.

Further investigation of the crystalline quality of the DH NM is done by high resolution X-ray diffraction (XRD) after the NM is transferred (N-polar face up) onto a SiO_2_/Si handle wafer. Reciprocal space mapping (RSM) using the (105) diffraction is shown in Fig. 4(a). The vertical alignment of AlGaN and GaN diffraction points confirm that both the top and bottom AlGaN layers remain pseudomorphic with the GaN layer^40^. Figure 4(b) shows (002) ω/2θ scan (radial scan) where the main peak and the broad shoulder peak correspond to GaN and AlGaN, respectively. The presence of Pendellösung fringes suggests atomically smooth interfaces and surfaces. According to the spacing of fringes, the thickness of GaN and AlGaN agrees with design values. The (002) and (102) diffraction rocking curves of the GaN layer are shown in Fig. 4(c) and (d). The full width at half maximum (FWHM) is 0.13° for (002) and 0.12° for (102). The slight increase of (002) FWHM is probably due to wrinkles of NM or non-uniform adhesion between NM and the wafer^18^. The screw dislocation density is measured by the Williamson-Hall method by fitting FWHM of GaN (002), (004) and (006) rocking curves. (Figure S3) The obtained value is 4.9 × 10^8^ cm^−2^, which fits with standard GaN epilayers grown on sapphire. According to the XRD results, AlGaN/GaN/AlGaN NM retains the crystalline quality of as-grown epitaxial structure with no degradation from the electrochemical lift-off process.

The strain can be calculated from measured lattice parameters. For the GaN layer in the NM, the c is 0.5187 nm obtained from (002), (004) and (006) ω/2θ scan. (Figure S4) Using c of 0.5185 nm for freestanding GaN and Poisson’s ratio of 0.183^41^, there is 0.1% in-plane compressive strain in the GaN layer induced by both Al_0.25_Ga_0.75_N layers, consistent with the Raman measurement.

In conventional AlGaN/GaN heterostructures on epitaxial wafers, the formation of 2DEG comes from both spontaneous and piezoelectric polarizations in the AlGaN layer. In the AlGaN/GaN/AlGaN nanomembrane configuration, 2DEG is also expected at the top hetero-interface, because the AlGaN layer is tensilely strained (=0.5%) according to micro-Raman and XRD measurement. Hall measurement on a 0.5 × 0.5 cm^2^ NM shows that, the 2DEG density in NM is 5.4 × 10^12^ cm^−2^, and the electron mobility is 790 cm^2^/V s. (Figure S5) Considering the reduced strain of the AlGaN layer after NM lift-off and the simplified structure without AlN spacing layer, the values are in agreement with previous results from AlGaN/GaN on sapphire^42^. This mobility is among the highest values reported for single-crystalline semiconductor NMs, and it surpasses most single crystal NMs, for example, Si and GaN^9, 31^. Still, It is likely that the 2DEG is affected by memory effect of Fe doped GaN. Further improvement of 2DEG can be made by replacing Fe-GaN with a carbon doped high resistive GaN layer and adding an AlN spacer. (Supplementary Information).

To complete the proof-of-concept of heterointegration, NM HEMT is fabricated on SiO_2_. The device processing starts with lifting off the AlGaN/GaN/AlGaN NM and transferring it onto SiO_2_/Si substrate with the Ga-polar surface facing up. A shallow ICP etching was conducted (depth = 50 nm) to create mesas with isolated 2DEG layers. Second, Ti/Al/Ni/Au was deposited as source and drain contact metal by evaporation and lift-off, and subsequently annealing at 800 °C for 1 min in N_2_ ambient. Later, 50 nm silicon nitride is deposited by PECVD to passivate and anchor the entire NM. Via holes for gate metal were opened by RIE etching, and Ni/Au is deposited as Schottky gate metal. Both via and gate were patterned by E-beam lithography. At last, Ni/Au metal pad is deposited.

**Figure 5:**
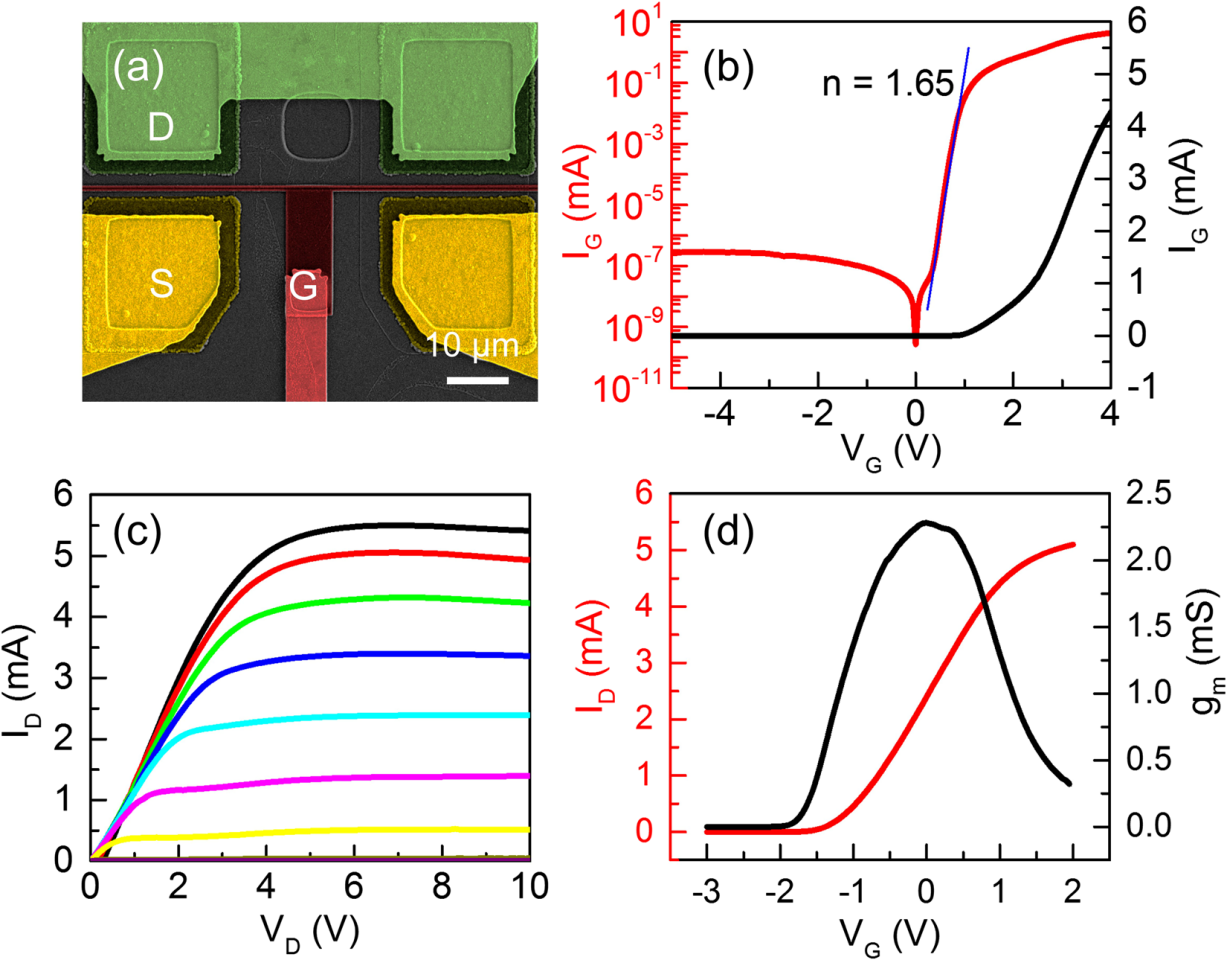
NM HEMT on SiO_2_/Si. (**a**) The SEM image of a device with two separate 25 µm × 4 µm channels. Electrodes are colored and labeled. I-V is measured with one channel. (**b**) I_G_-V_G_ of the Schottky gate in linear (black) and logarithmic (red) scales. (**c**) I_D_-V_D_ at different gate bias from −3 V to 2 V, with step of 0.5 V. (**d**) Transfer characteristics (I_D_-V_G_) with V_D_ of 4 V (red curve). Measured transconductance g_m_ is also shown in black.

The finished device is shown in Fig. 5(a). The channel is 25 µm × 2 in width and 4 µm in length. The gate length is 250 nm. For all the measurements in Fig. 5(b)–(d), only one side of the channel is used. I-V of Schottky gate diode is shown in Fig. 5(b). The reverse current at −4 V is 2.8 × 10^−7^ mA and forward current at 4 V is 4.2 mA, with rectification ratio of over 10^7^. The ideality factor is 1.65. The results suggest that the AlGaN/GaN NM is of high crystalline quality with negligible leakage paths. The current-voltage characteristics are shown in Fig. 5(c) and (d). The current density and transconductance is 200 mA/mm and is 90 mS/mm, respectively. We note that this transconductance is a factor of 3 to 4 lower than the conventional AlGaN transistors on sapphire, mainly due to the low-Al content and low 2DEG concentration. Strategy in increasing 2DEG in the AlGaN NM is currently under way.

**Figure 6:**
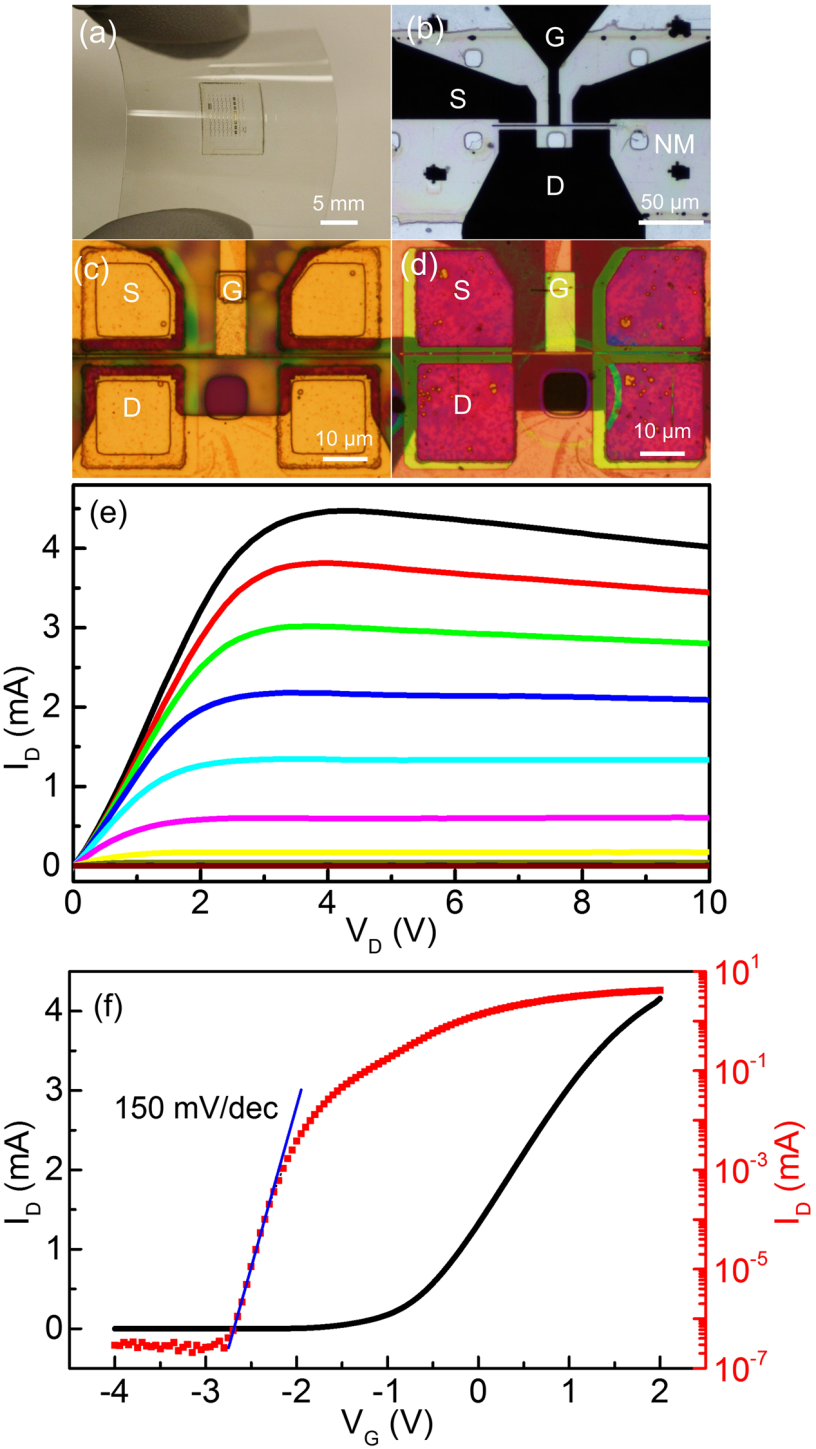
NM HEMT on polyester substrate. (**a**) Photo of NM HEMTs on PET with bend radius of about 3 cm. (**b**) NM HEMT microscopy image with back illumination, showing the transparency of the NM. (**c**) Front side of active region. (**d**) Backside of active region. (**e**) I_D_-V_D_ with gate bias from −3 V to 2 V, with step of 0.5 V. (**f**) I_D_-V_G_ with V_D_ of 4 V.

The fabrication of NM HEMT on flexible, polymeric templates requires further procedural refinement due to a much lower thermal budget. Conventional ohmic contacts to source and drain need a high annealing temperature (usually >750 °C) which is far above the melting point of PET. An additional constraint arises since during the alloying process on thick epilayer structures, metals could diffuse into GaN for over a hundred nanometers^43^, thus altering the electrochemical etching process. The alloyed contacts are often corroded during EC etching, making it not feasible to make ohmic contacts prior to electrochemical etching. As a consequence, the NM HEMTs are initially fabricated on SiO_2_/Si template. After the HEMT is fabricated, the NM HEMT/SiO_2_/Si structure is then flip-chip bonded onto a PET film using SU-8 as an adhesive. Then, the Si substrate is selectively etched away by gas phase XeF_2_. During the substrate etching, the NM device are protected by SiO_2_ due to a very high etching selectivity of Si over SiO_2_ (likely >10000:1). Finally, the SiO_2_ is fully removed to expose the metal pads. (Figure S6) Fig. 6(a)–(d) show the images of the device on the 250 µm-thick PET. When back illuminated, the active region of the device is optically transparent, with gate metal visible under microscope.

I_D_-V_D_ and I_D_-V_G_ characteristics of the device are shown in Fig. 6(e) and (f). Comparing to NM HEMTs on SiO_2_/Si, the threshold shifts to a positive direction by around 0.3 V, probably due to thermal strain induced by polymers. Despite of thermal dissipation problem of PET, the ON/OFF ratio of the device is 10^7^, the sub-threshold swing of transconductance is 150 mV/decade. The parameters compared very favorably with other NM transistors^1, 9, 31^. Finally, there is apparent current drop for HEMTs on PET films with increasing V_D_ after saturation (Fig. 6(e)), likely due to heating effect. We speculate that the channel temperature has increased during operation as a heating power of over 40 mW is generated within 25 µm × 4 µm channel region. (Figure S7) At an elevated temperature, resistance of the NM rises from a reduced mobility, reducing effectively V_G_ and g_m_, respectively^44^. Considering the glass transition of PET film begins at 80 °C and its large thermal expansion coefficient^45^, both heat and strain contribute to affect device performance. While the potential mechanisms are identified, the thermal dissipation can further be enhanced by employing a heat dissipation layer such as UNCD or Graphene^46, 47^.

## Discussion

In summary, AlGaN/GaN high mobility transistors in the form of a nanomembrane (~300 nm) have been fabricated. The NM invokes a double-heterostructure design to achieve strain balancing. The crystalline quality of freestanding AlGaN/GaN NM, with dislocation about 5 × 10^8^ cm^−2^, is well preserved throughout the fabrication process. Transport measurement shows that 2DEG density is 5.4 × 10^12^ cm^−2^ with a mobility of 790 cm^2^/Vs. AlGaN/GaN NM HEMTs are fabricated on both rigid and flexible polymeric substrates. The ON/OFF ratio of the device is greater than 10^7^, with a sub-threshold swing of 150 mV/dec. The study shows that III-Nitrides NM is a promising candidate for high performance electronics.

## Methods

### Material growth and lift-off

The samples were grown in a horizontal metal-organic chemical vapor deposition (MOCVD) reactor (Aixtron 200/4 RF-S) on 2″ c-plane sapphire substrates. Trimethyl gallium (TMGa), trimethyl aluminum (TMAl), and ammonia (NH_3_) were used as gallium (Ga), indium (Al), and nitrogen (N) source material, respectively. Silane (SiH_4_) was used for n-type doping source. After growth, the samples were lithographically patterned with photoresist Shipley 1827; and via holes were etched by inductively coupled plasma (ICP) reactive-ion etching in an Oxford 100 chamber to expose the sidewalls of the highly-doped layer. Subsequently, the AlGaN/GaN/AlGaN double heterostructure nanomembrane (NM) was lifted off from the substrate by electrochemically etching the n++ GaN sacrificial layer using a Keithley 2400 as the voltage source. After lift-off, the NM was transferred onto Si for characterization. Micro Raman mapping was performed by Raman Microscope Thermo Scientific DXRxi using 532 nm laser source and 50× object lens. The crystal characterization was done by PANalytical X’Pert PRO X-ray diffractometer.

### Fabrication of HEMT devices

For HEMT on SiO_2_, first, device isolating mesa was created with a height of 50 nm by ICP in a PlasmaTherm 790 etching system. Next, Ti/Al/Ni/Au was deposited as source and drain contacts by CHA 600 E-beam Evaporator, and annealed at 800 °C for 1 min in N_2_ ambient. 50 nm silicon nitride was then deposited by plasma-enhanced chemical vapor deposition in PlasmaTherm 70 at 250 °C. The window for gate contact was opened by RIE etching in Unaxis 790, and Ni/Au is deposited as the gate metal. Both the window and gate metal were patterned by E-beam lithography. Lastly, Ni/Au was used as contact pads. For HEMT on PET film, the device on SiO2/Si was bonded with PET film using SU-8 as the agent. Then the Si substrate was removed from the SPTS Xactix e1 Xenon Difluoride (XeF_2_) Etcher. After fabrication, DC characteristics of the devices were measured with an Agilent 4155 semiconductor parameter analyzer.

## Electronic supplementary material

Supplementary Information
