# Supplementary material for: Strain Balanced AlGaN/GaN/AlGaN nanomembrane HEMTs
Source: Sci Rep. 2017 Jul 25;7:6360. doi: 10.1038/s41598-017-06957-8 (PMC5527108; doi:10.1038/s41598-017-06957-8)
Supplement: Supplementary file 1 — Supplementary Information [file 41598_2017_6957_MOESM1_ESM.pdf]

Strain Balanced AlGaN/GaN/AlGaN nanomembrane HEMTs

Tzu-Hsuan Chang[1], Kanglin Xiong[2,], Sung Hyun Park[2], Ge Yuan[2], Zhenqiang Ma[1], and Jung Han[2]*

[1]Department of Electrical and Computer Engineering, University of Wisconsin-Madison, Madison, 53706, United States

[2]Department of Electrical Engineering, Yale University, New Haven, 06511, United States

[*]kanglin.xiong@yale.edu

Nanomembrane fabrication

The preparation of nanomembrane (NM) begins with the epitaxial growth of III-Nitrides on Sapphire substrate by MOCVD. The detailed structure is shown in Figure S1 (a). The structure can be categorized into three parts according to their function from bottom to top, that is, the buffer and current spreading layer, the sacrificial layer, and the NM to be lift-off. During electrochemical etching, the heavily doped n^{++} GaN will be etched, while other layers are intact. The current spreading layer enable current to flow across the whole wafer laterally, so that the sacrificial layer can be fully and uniformly etched. The electrolyte for this experiments is a mixture of HF (49%), Ethanol and Glycerol in the volume ratio of 1: 2: 3. The bias voltage for electrochemical etching is 25 V.[1] To apply the voltage to the sample, one corner of the sample is contacted with indium

and connected to the anode of voltage source. A platinum wire is used as cathode. The etching front is shown in Figure S1 (b).

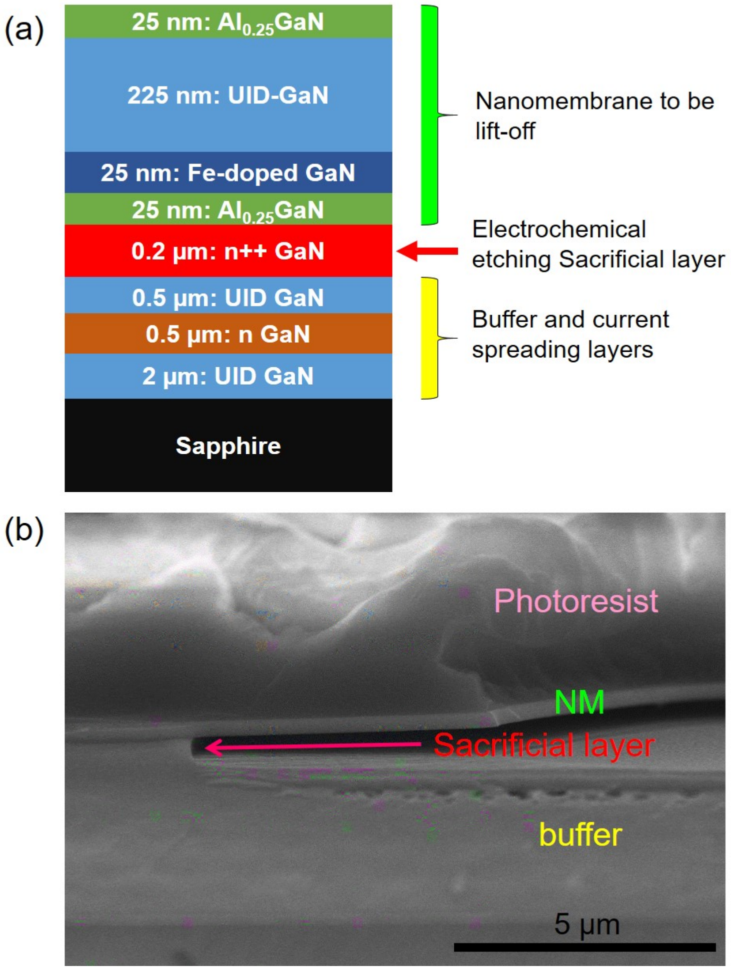

Figure S1. (a) Schematic diagram of epitaxial structure for NM lift-off. The layers are categorized according to their functions. (b) SEM image of the sample after partial undercut etching of n^{++} GaN sacrificial layer.

Two challenges (parasite etching of 2DEG, NM buckling)

The EC etching of n^{++} sacrificial layer is conductivity selective, and also relies on the current flow path within the sample. In initial experiments, it is noticed that the top AlGaN/GaN interface with

2DEG is attacked as shown in Figure S2 (a), due to its high electron concentration and mobility. A high resistive Fe-doped GaN has been used to prevent the vertical current flow into the 2DEG, which in turn stops the EC etching of the interface.

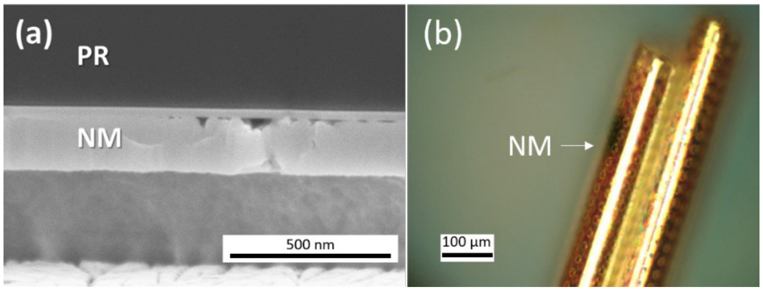

Figure S2. (a) Cross sectional SEM image of a sample without Fe-doped GaN after electrochemical etching. The 2DEG region has been etched. (b) Microscope image of a freestanding AlGaN/GaN NM. The NM curled up into cylindrical shape due to unbalanced strain.

Freestanding $Al_{0.25}Ga_{0.75}N$/GaN NM will curl up as shown in Figure S2 (b) due to unbalanced strain. The strain of AlGaN layer (ϵ_{AlGaN}) depends on its Al content (x_{Al}), and can be calculated from the 2.4% lattice mismatch between AlN and GaN and the strain partition rule.

$$\epsilon_{AlGaN} = 2.4\%\, x_{Al} \frac{h_{GaN}}{h_{AlGaN}+h_{GaN}} \quad \text{(S1)}$$

Where h_{GaN} and h_{AlGaN} is the thickness of GaN and AlGaN layer in the NM, respectively.

The radius of curvature (R) is inversely proportional to the strain of AlGaN layer (ϵ_{AlGaN}), described by the Stoney equation.[2]

$$\epsilon_{AlGaN} = \frac{h_{GaN}^2}{6(1-\nu)h_{AlGaN}} \frac{1}{R} \quad \text{(S2)}$$

Where v is the Poisson ratio of GaN (0.183). The calculated R is 35.7 µm, consistent what is shown in Figure S2 (b).

By cancelling ϵ_{AlGaN} in the above two equations, the dependence of R on x_{Al} can be expressed as,

$$R = \frac{h_{GaN}(h_{GaN}+h_{AlGaN})}{6(1-\nu)h_{AlGaN}} / 2.4\% \, x_{Al} \quad \text{(S3)}$$

Then R = 8.9 µm / x_{Al} is obtained for h_{GaN} = 150 nm and h_{AlGaN} = 25 nm.

By using AlGaN/GaN/AlGaN structure, lift-off NMs remain flat. The final structure is shown in Figure S1 (a), where the thickness of the sandwiched GaN layer has been increased to 250 nm to keep the strain in AlGaN layer and 2DEG.

Dislocation density of freestanding NM

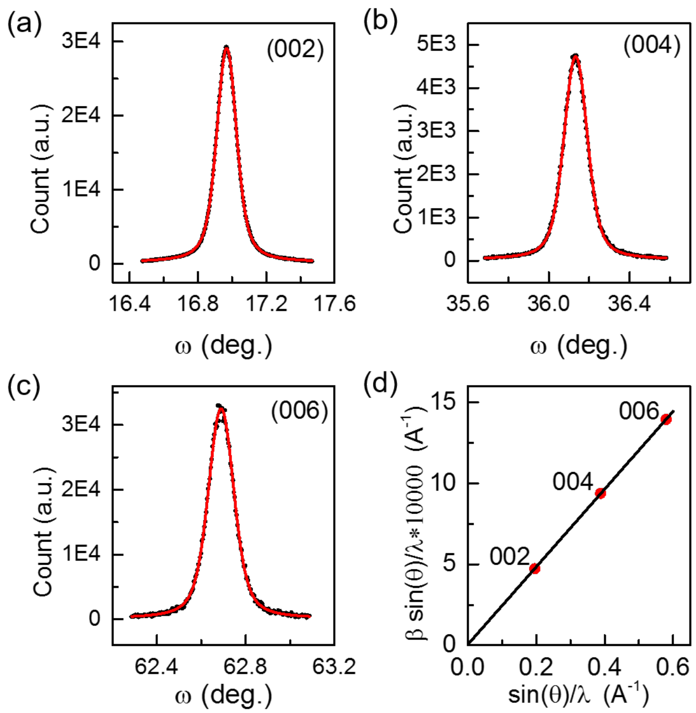

Figure S3. Rocking curves of GaN layer within NM (a) (002), (b) (004) and (c) (006). (d) Williamson-Hall plot.

X-ray diffraction can measure the screw dislocation density within the NM.[3] The screw dislocation density ρ_{sc} can be calculated as,

$$\rho_{sc} = \alpha_{\omega}^{2}/2\pi \ln(2)\, b_{sc}^{2} \quad \text{(S4)}$$

Where b_{sc} is norm of Burgers vector of (100) screw dislocation, which is 0.5185 nm for GaN. And α_ω is the tilt angle that can be obtained from the slope of Williamson-Hall Plot as,

$$\alpha_{\omega} = \arctan(slope) \quad \text{(S5)}$$

The Williamson-Hall plot is ($\beta \sin(\theta)/\lambda$) versus $\sin(\theta)/\lambda$ at different Bragg angles θ for (002), (004) and (006), where β is FWHM of ω-scan. And λ is X-ray wavelength.

In the measurement, the ω-scan is performed in symmetrical configuration. The results is shown in Figure S3. The obtained α_ω is 0.0024, which corresponds to screw dislocation density of 4.9×10^{8} cm^{-2}.

Strain of the sandwiched GaN layer

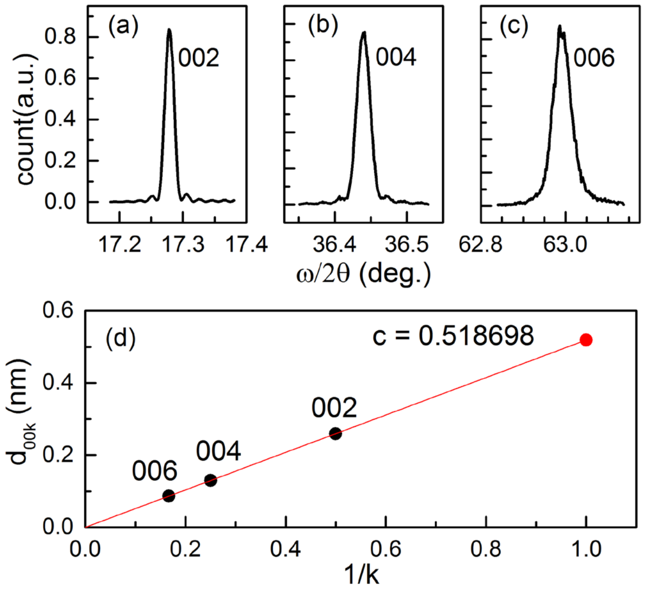

Figure S4. (a)-(c) The (002), (004) and (006) XRD ω/2θ scan of the GaN layer in the AlGaN/GaN/AlGaN NM. (d) The calculation of lattice c of GaN by linear fitting of lattice spacing d_{00k} of different (00k) planes. The vertical coordinate of red dot corresponds to the c parameter.

The strain can be calculated from lattice parameter. For the GaN layer, the lattice c is 0.5187 nm extracted from XRD ω/2θ scans shown in Figure S4 (a)-(c). Assuming c of 0.5185 nm for freestanding GaN and Poisson ratio of 0.183,[4] the in-plane compressive strain of GaN is 0.1%.

Temperature dependent Hall measurement

The electrical properties of 2DEG at top AlGaN/GaN interface of the NM is measured by temperature-dependent Hall method. The sample under test is a strain balanced NM (5 × 5 mm^2) transferred onto SiO_2/Sapphire. Regarding to the arrays of via holes in the NM, the mobility will be slightly underestimated while the electron concentration will be accurate.[5] The results are shown in in Figure S5. The sheet resistance increases in nonlinear way with temperature, which can be

fitted using Callender–Van Dusen equation.[6] The decrease of electron mobility is expected because of increased phonon scattering at higher temperature. The sheet electron density change will affect the threshold of NM HEMT.

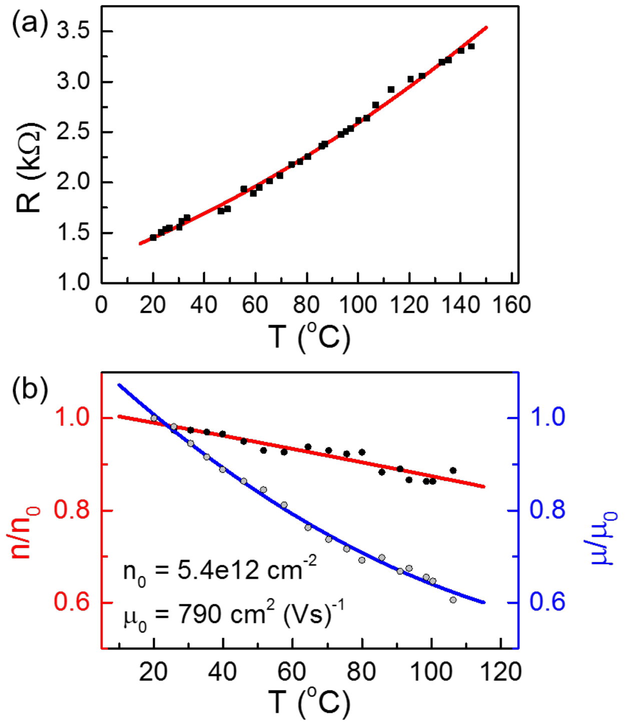

Figure S5. Temperature dependent (a) sheet resistance of 2DEG at top AlGaN/GaN interface of NM, The red line is fitted using Callender–Van Dusen equation. (b) Sheet carrier density and mobility normalized by values measured at room temperature. The lines are for eyes only.

The 2DEG can be improved in several ways. First, Fe-doping of GaN has memory effect which means the profile of Fe cannot turned off sharply. The concentration of Fe decay approximately exponentially with a characteristic length of about 200 nm.[7] So it is likely that the 225 nm uid-GaN in the NM is not thick enough to eliminate the influence of Fe. Carbon doped GaN does not have memory effect, and can be used as high resistive layer instead of Fe-GaN to boost electron

density and mobility.[8] Second, in current NM, no AlN spacer layer is used. Mobility is compromised by alloy scattering. A thin AlN (~ 1 nm) layer can be inserted in between AlGaN and uid-GaN to reduce alloy scattering from AlGaN.

Fabrication of NM HEMT/ PET

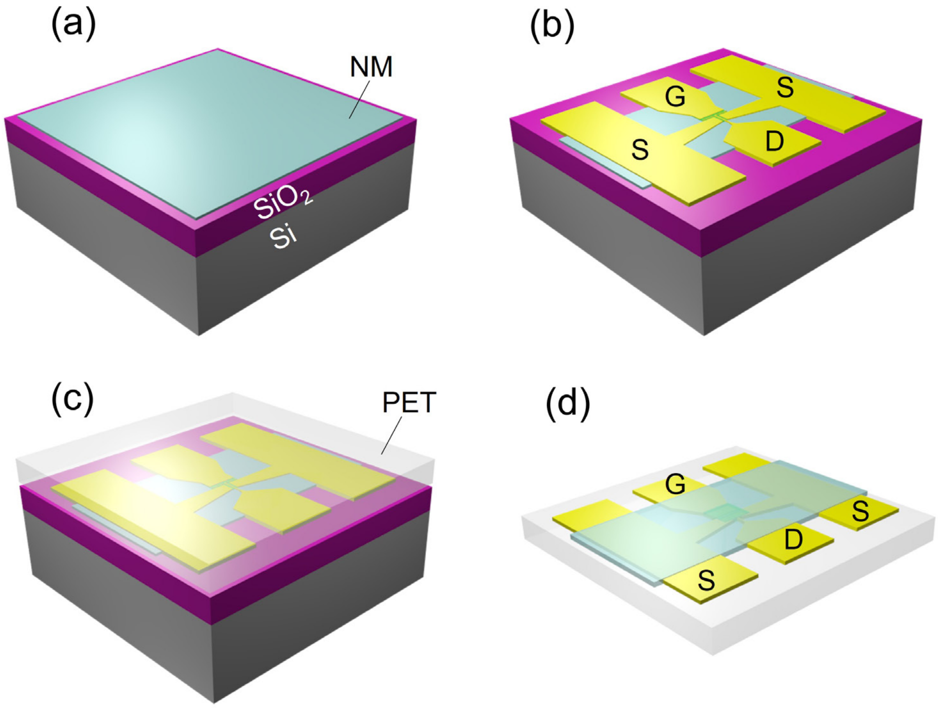

Figure S6. Schematic diagram of NM HEMT/PET fabrication flow. (a) Ga-polar NM transferred onto SiO_2/Si. (b) NM is patterned into stripes, and HEMT is fabricated using conventional processing techniques. (c) During the transfer of HEMT onto PET, the Si wafer is flip-chip bonded with a PET film. (d) After removal of the Si and SiO_2, the NM is on PET with exposed metal pads ready for measurement.

For NM HEMT on PET, the device is first fabricated on SiO_2/Si substrate, then transferred onto PET. (Figure S6) The following is transfer procedure.

1) Spin SU-8 2000.5 on the NM HEMT/SiO_2/Si at 4000 rpm. Bake the wafer at 180 ºC for 5 min on hotplate.

2) Put a drop of SU-8 2002 on to the sample surface. Flip-chip bond the sample onto a piece of PET film.

3) Bake the bonded Si/SiO_2/NM HEMT/SU-8/PET sample at 90 ºC for 2 hours. Apply pressure to the Si against PET during the backing.

4) Flood expose the sample in mask aligner to cure the SU-8 for permanent bonding of NM HEMT with PET.

5) Remove the Si substrate by fully etching it in a pulsed XeF_2 etcher. The left structure will be SiO_2/NM HEMT/SU-8/PET.

6) RIE etch SiO_2 to expose the metal pads of NM HEMTs.

The resulted NM HEMT/SU-8/PET is ready for measurement. Because the chip is flipped when bonding to PET film, the active region of the HEMT is sandwiched between the NM and PET film.

Heat dissipation of NM HEMT/PET

According to simulation, Figure 7S shows that the channel temperature of NM HEMT on PET film increases with input power. The spatial non-uniformity of temperature distribution is obvious. Both the high temperature and the non-uniformity will influence the device performance.

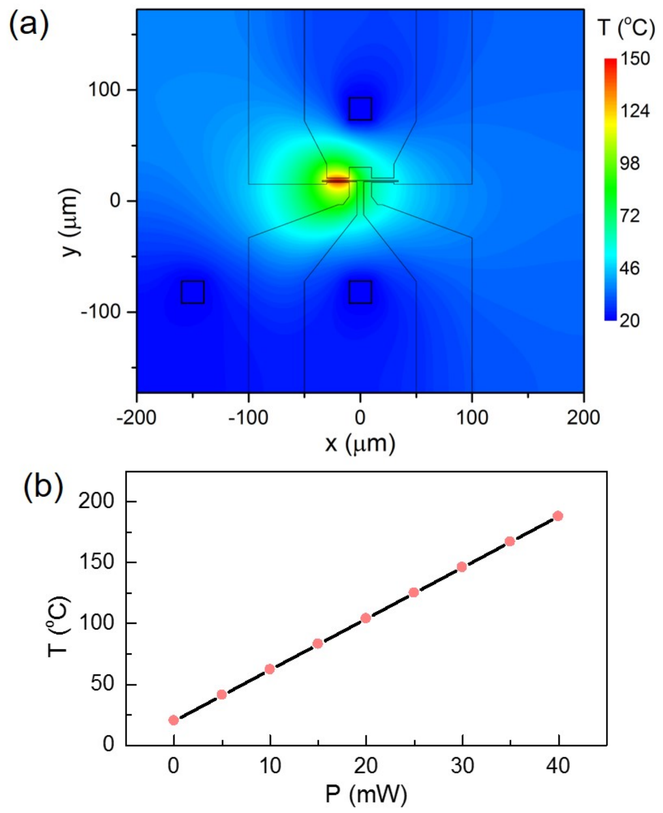

Figure S7. (a) Simulated static temperature distribution of NM HEMT powered at 30 mW. The three squares are in contact with electrical probe tips at 20 °C. (b) Simulated channel temperature of NM HEMT increases with power. For all the simulation, the metal pads are 300 nm thick gold, the thermal conductivity of NM is 230 W/K m.

Ignoring the change in electron saturation velocity υ_{sat} and leakage from substrate, the change in saturated current I_{sat} can be expressed as,[9]

$$\Delta I_{sat} = -g_m (I_{sat} \Delta R_S + \Delta V_T) \tag{S6}$$

Where R_S is the sum of the contact resistance and the resistance of the channel between the source and gate contacts. V_T is the threshold voltage, which is affected by the sheet carrier density and Schottky barrier height.

When temperature rises, the saturated current I_{sat} is affected by the drop of effective gate bias $I_{sat}\Delta R_S$ as well as the shifting of threshold voltage ΔV_T. Decrease of I_{sat} has been observed for NM HEMT on PET with increased power.

Besides, due to the high temperature as well as its non-uniformity, the PET substrate will be under dramatic change such as expansion, melting or glass transition. The NM HEMT will be mechanically strained by the deformation of PET. The strain will affect the 2DEG by piezoelectricity and eventually device performance.

It is noticed that the thermal budget of NM HEMT/PET is limited by the low thermoconductivity of the PET film. To improve the heat dissipation, substrate with high thermal conductivity can be used. So heat can then be laterally spread out, and dissipated through surrounding air or heat sinks.

References

1. Chen, D., Xiao, H. & Han, J. Nanopores in GaN by electrochemical anodization in hydrofluoric acid: Formation and mechanism. *J. Appl. Phys.* **112,** 064303 (2012).
2. Janssen, G. C. A. M., Abdalla, M. M., van Keulen, F., Pujada, B. R. & van Venrooy, B. Celebrating the 100th anniversary of the Stoney equation for film stress: Developments from polycrystalline steel strips to single crystal silicon wafers. *Thin Solid Films* **517,** 1858–1867 (2009).
3. Metzger, T. *et al.* Defect structure of epitaxial GaN films determined by transmission electron microscopy and triple-axis X-ray diffractometry. *Philos. Mag. A* **77,** 1013–1025 (1998).

4. Moram, M. A., Barber, Z. H. & Humphreys, C. J. Accurate experimental determination of the Poisson's ratio of GaN using high-resolution x-ray diffraction. *J. Appl. Phys.* **102,** 023505 (2007).
5. Xiong, K. *et al.* Single Crystal Gallium Nitride Nanomembrane Photoconductor and Field Effect Transistor. *Adv. Funct. Mater.* **24,** 6503–6508 (2014).
6. Zahmani, A. H. *et al.* Temperature Dependence of the Resistance of AlGaN/GaN Heterostructures and Their Applications as Temperature Sensors. *Jpn. J. Appl. Phys.* **49,** 04DF14 (2010).
7. Heikman, S., Keller, S., DenBaars, S. P. & Mishra, U. K. Growth of Fe doped semi-insulating GaN by metalorganic chemical vapor deposition. *Appl. Phys. Lett.* **81,** 439–441 (2002).
8. Gamarra, P. *et al.* Optimisation of a carbon doped buffer layer for AlGaN/GaN HEMT devices. *J. Cryst. Growth* **414,** 232–236 (2015).
9. Kuzmik, J. *et al.* Determination of channel temperature in AlGaN/GaN HEMTs grown on sapphire and silicon substrates using DC characterization method. *IEEE Trans. Electron Devices* **49,** 1496–1498 (2002).
